# The Phylogeny of Little Red Riding Hood

**DOI:** 10.1371/journal.pone.0078871

**Published:** 2013-11-13

**Authors:** Jamshid J. Tehrani

**Affiliations:** Department of Anthropology and Centre for the Coevolution of Biology and Culture, Durham University, Science Site, South Road, Durham, United Kingdom; Bristol University, United Kingdom

## Abstract

Researchers have long been fascinated by the strong continuities evident in the oral traditions associated with different cultures. According to the ‘historic-geographic’ school, it is possible to classify similar tales into “international types” and trace them back to their original archetypes. However, critics argue that folktale traditions are fundamentally fluid, and that most international types are artificial constructs. Here, these issues are addressed using phylogenetic methods that were originally developed to reconstruct evolutionary relationships among biological species, and which have been recently applied to a range of cultural phenomena. The study focuses on one of the most debated international types in the literature: ATU 333, ‘Little Red Riding Hood’. A number of variants of ATU 333 have been recorded in European oral traditions, and it has been suggested that the group may include tales from other regions, including Africa and East Asia. However, in many of these cases, it is difficult to differentiate ATU 333 from another widespread international folktale, ATU 123, ‘The Wolf and the Kids’. To shed more light on these relationships, data on 58 folktales were analysed using cladistic, Bayesian and phylogenetic network-based methods. The results demonstrate that, contrary to the claims made by critics of the historic-geographic approach, it is possible to identify ATU 333 and ATU 123 as distinct international types. They further suggest that most of the African tales can be classified as variants of ATU 123, while the East Asian tales probably evolved by blending together elements of both ATU 333 and ATU 123. These findings demonstrate that phylogenetic methods provide a powerful set of tools for testing hypotheses about cross-cultural relationships among folktales, and point towards exciting new directions for research into the transmission and evolution of oral narratives.

## Introduction

The publication of Jacob and Wilhelm Grimm's *Children's and Household Tales* (1812–1814) [1] two hundred years ago sparked enormous public and academic interest in traditional stories told among “the common people”, and helped establish folklore as a field for serious academic inquiry. Inspired by the Grimms' methods, a new generation of researchers ventured outside the library and into the villages and households of the rural peasantry to collect colourful tales of magical beasts, wicked stepmothers, enchanted objects, and indefatigable heroes [2]. One of the most unexpected and exciting discoveries to emerge from these studies was the recurrence of many of the same plots in the oral traditions associated with different – and often widely separated – societies and ethnic groups. Thus, the Brothers Grimm noted that many of the ostensibly “German” folktales which they compiled are recognisably related to stories recorded in Slavonic, Indian, Persian and Arabic oral traditions [3]. These similarities have attracted the attention of folklorists, literary scholars, anthropologists, cognitive scientists and others for a variety of reasons: For example, cognate tales in other cultures have been studied to try and reconstruct the origins and forms of classic western fairy tales before they were first written down [2] [4]. Other researchers have examined the distributions of common plot elements within and across regions to make inferences about past migration, cross-cultural contact, and the impact of geographical distance and language barriers on cultural diffusion [5] [6]. Last, it has been suggested that patterns of stability and change in stories can furnish rich insights into universal and variable aspects of the human experience, and reveal how psychological, social and ecological processes interact with one another to shape cultural continuity and diversity [7] [8] [9].

Unfortunately, since folktales are mainly transmitted via oral rather than written means, reconstructing their history and development across cultures has proven to be a complex challenge. To date, the most ambitious and sustained effort in this area has been carried out by folklorists associated with the so-called “historic-geographic” school, which was established toward the end of the nineteenth century [10]. These researchers have sought to classify similar folktales from different oral literatures into distinct “international types” based on consistencies in their themes, plots and characters. The most comprehensive and up-to-date reference work in this field, the Aarne-Uther-Thompson (ATU) index, identifies more than two thousand international types distributed across three hundred cultures worldwide [11]. Exponents of the historic-geographic school believed that each international type could be traced back to an original “archetype” tale that was inherited from a common ancestral population, or spread across societies through trade, migration and conquest. Over time, the tales' original forms were then adapted to suit different cultural norms and preferences, giving rise to locally distinct “ecotypes” [5]. The historic-geographic method sought to reconstruct this process by assembling all the known variants of the international type and sorting them by region and chronology. Rare or highly localised forms were considered to be of likely recent origin, whereas widespread forms were believed to be probably ancient, particularly when they were consistent with the earliest recorded versions of the tale [2] [4].

The historic-geographic method has been criticised for a number of reasons [4]. First, it has been suggested that the criteria on which international types are based are arbitrary and ethnocentric. Most types are defined by the presence of just one or two plot features (“motifs”), and gloss over dissimilarities among tales within the same group as well as their resemblances to tales belonging to other groups [12]. Since the majority of international types were originally defined in relation to the western corpus, tales from other regions are often difficult to classify according to the ATU index because they lack one or more of the key diagnostic motifs, or fall between supposedly distinct international types [12] [13]. A second, related problem with the historic-geographic method is sampling bias. Given that European folklore traditions have been studied far more intensively than any others, reconstructions based on the frequency and chronologies of variants are likely to be heavily skewed. Last of all, some researchers have suggested orally transmitted tales are too fluid and unstable to be classified into distinct groups based on common descent, and that the classification of international types is often superficial [13] [14]. According to this view, the aims of the historic-geographic school are at best unrealistic, if not entirely misconceived.

This study proposes a novel approach to studying cross-cultural relationships among folktales that employs powerful, quantitative methods of phylogenetic analysis. Phylogenetics was originally developed to investigate the evolutionary relationships among biological species, and has become increasingly popular in studies of cultural phenomena (dubbed “phylomemetics” [15]), including languages [16] [17] [18] [19] [20] [20], manuscript traditions [21] [22] [23] and material culture assemblages [24] [25] [26] [27] [28] [29] [30] [25] [31]. In each case, the aim of a phylogenetic analysis is to construct a tree or graph that represents relationships of common ancestry inferred from shared inherited traits (homologies). Folktales represent an excellent target for phylogenetic analysis because they are, almost by definition, products of descent with modification: Rather than being composed by a single author, a folktale typically evolves gradually over time, with new parts of the story added and others lost as it gets passed down from generation to generation. Recent case studies of the urban legend ‘Bloody Mary’ [32], the ‘Pygmalion’ family of myths in Africa [33], and western European variants of the folktale ‘The Kind and the Unkind Girls’ [6] have demonstrated the utility of phylogenetic techniques for reconstructing relationships among variants within a given tale type. The present study aims to establish whether these methods can also be used to differentiate the tale types themselves, and test the empirical validity of the international type system.

In addressing this question, phylogenetics has several advantages over traditional historic-geographic methods. First, rather than basing the classification of related tales on just a few privileged motifs, phylogenetic analysis can take into account all the features that a researcher believes might be relevant. Second, phylogenetic reconstruction does not assume a-priori that the most common form of a trait, or the form exhibited by the oldest recorded variant, is necessarily ancestral. It is therefore likely to be less vulnerable to the strong European bias in the folktale record than traditional historic-geographic methods. Third, phylogenetics provides useful tools for quantifying the relative roles of descent versus other processes, such as convergence and contamination, in generating similarities among taxa. These include statistical techniques for measuring how well patterns in a dataset fit a tree-like model of descent [34], and network-based phylogenetic methods that have been designed to capture conflicting relationships [35], [36]. Such methods make it possible to evaluate the coherence and degree of overlap between international types indicated by the analyses.

The study focuses on one of the most famous and controversial international types in the folktale literature, ATU 333 – ‘Little Red Riding Hood’ [37] [38]. Most versions of the story in modern popular culture are derived from the classic literary tale published by Charles Perrault in seventeenth century France [39], which recounts the misadventures of a young girl who visits her grandmother's house, where she is eaten by a wolf disguised as the old woman. It is widely believed that Perrault based his text on an old folktale known simply as ‘The Story of Grandmother’, versions of which have survived in the oral traditions of rural France, Austria and northern Italy [38]. In many of these tales, the girl lacks her characteristic red hood and nickname, and manages to outwit the wolf before he can eat her: After finally seeing through the villain's disguise, the girl asks to go outside to the toilet. The wolf reluctantly agrees, but ties a rope to her ankle to prevent her from escaping. When she gets out, the girl cuts the rope, ties the end to a tree, and flees into the woods before the villain realises his mistake. Another variant of the plot has the young girl – commonly named Catterinella – taking a basket of cakes to her aunt/uncle, who turns out to be a witch or werewolf. On the way there, she eats the cakes and replaces them with donkey dung. When the aunt/uncle discovers her deception, (s) he comes to her house at night and devours her in bed. Although these tales were recorded long after Perrault published his version, a rediscovered 11^th^ century poem written in Latin by a priest in Liège provides intriguing evidence that a story similar to Little Red Riding Hood was circulating in parts of western Europe in medieval times [40]. The poem, which purports to be based on a local folktale, tells of a girl who wanders into the woods wearing a red baptism tunic given to her by her godfather. She encounters a wolf, who takes her back to its lair, but the girl manages to escape by taming the wolf's cubs.

Highly similar stories to Little Red Riding Hood have been recorded in various non-western oral literatures. These include a folktale that is popular in Japan, China, Korea and other parts of East Asia known as ‘The Tiger Grandmother’ [41] [42], in which a group of siblings spend the night in bed with a tiger or monster who poses as their grandmother. When the children hear the sound of their youngest sibling being eaten, they trick the villain into letting them outside to go to the toilet, where, like the heroine of The Story of Grandmother, they manage to escape. Another tale, found in central and southern Africa [43] [44], tells of a girl who is attacked by an ogre after he imitates the voice of her brother. In some cases, the victim is cut out of the ogre's belly alive – an ending that echoes some variants of Little Red Riding Hood recorded in Europe, including a famous text published by the Brothers Grimm in nineteenth century Germany [1].

Despite these similarities, it is not clear whether these tales can in fact be classified as ATU 333. Some writers [44] [45] [46] suggest they may belong to another international tale type, ATU 123, The Wolf and the Kids, which is popular throughout Europe and the Middle East. In this tale, a nanny goat warns her kids not to open the door while she is out in the fields, but is overheard by a wolf. When she leaves, the wolf impersonates her and tricks the kids into letting him in, whereupon he devours them. Versions of the tale occur in collections of Aesop's fables, in which the goat kid avoids being eaten by heeding the mother's instruction not to open the door, or seeks further proof of the wolf's identity before turning him away. In an Indian cognate of The Wolf and the Kids, known as ‘The Sparrow and the Crow’, the villain tricks the mother into letting her into the house, and eats her hatchlings during the night. Although ATU 123 is believed to be closely related to ATU 333, it is classified as a separate international tale type on the basis of two distinguishing features. First, ATU 333 features a single victim who is a human girl, whereas ATU 123 features multiple victims (a group of siblings) who are animals. Second, in ATU 333 the victim is attacked in her grandmother's house, while in ATU 123 the victims are attacked in their own home. However, the application of these criteria to non-western oral traditions is highly problematic: Thus, in most of the African tales the victim is a human girl (grouping them with ATU 333), but she is attacked in her own home rather than a relative's (grouping them with ATU 123). The East Asian tales also feature human protagonists (ATU 333), but they are usually a group of siblings rather than a single child (ATU 123). In most variants of the tale, they are attacked after being left at home by their mother (ATU 123), but in some cases they encounter the villain en route to their grandmother's house (as per ATU 333).

The ambiguities surrounding the classification of the East Asian and African tales exemplify the problems of current folklore taxonomy. While ATU 333 and ATU 123 are easy to discriminate between in a western context, tales from other regions share characteristics with both types and do not comfortably fit the definitions of either. With that in mind, the present study addresses two key questions: Can the tales described above be divided into phylogenetically distinct international types? If so, should the African and East Asian tales be classified as variants of ATU 333 or ATU 123?

**Figure 1:**
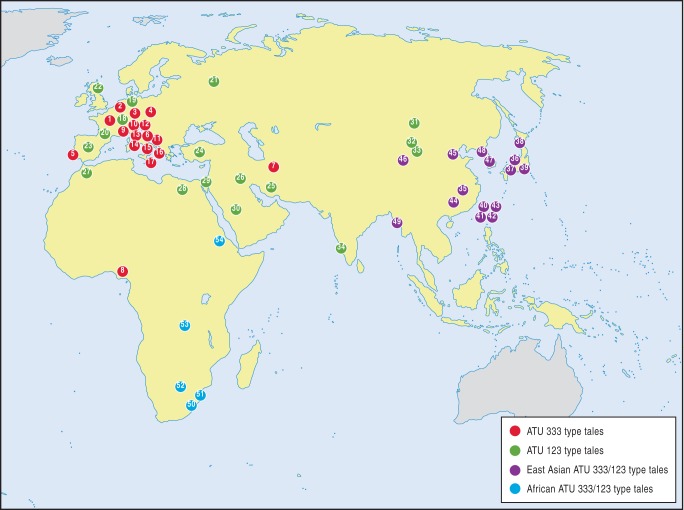
Map of the approximate locations from which tales were sourced. Numbers in the circles refer to the variants listed in the Supporting Information (Table SI.1).

Data for the study were drawn from 58 variants of ATU 333/123 available in English translation from 33 populations (listed in Table S1). The tales comprise a representative sample of the geographic distribution of ATU 333/123 type tales (Figure 1), and the plot variations described in regional tale-type and motif indicies [11] [41] [42] [44]. Relationships among the tales were reconstructed using three methods of phylogenetic analysis: cladistics, Bayesian inference and NeighbourNet (see Methods for a full description). The analyses focused on 72 plot variables, such as character of the protagonist (single child versus group of siblings; male versus female), the character of the villain (wolf, ogre, tiger, etc.), the tricks used by the villain to deceive the victim (false voice, disguised paws, etc.), whether the victim is devoured, escapes or is rescued, and so on. A full list of characters and explanation of the coding scheme is provided in the Supporting Information (File S1), together with the character matrix (File S2).

## Results

**Figure 2:**
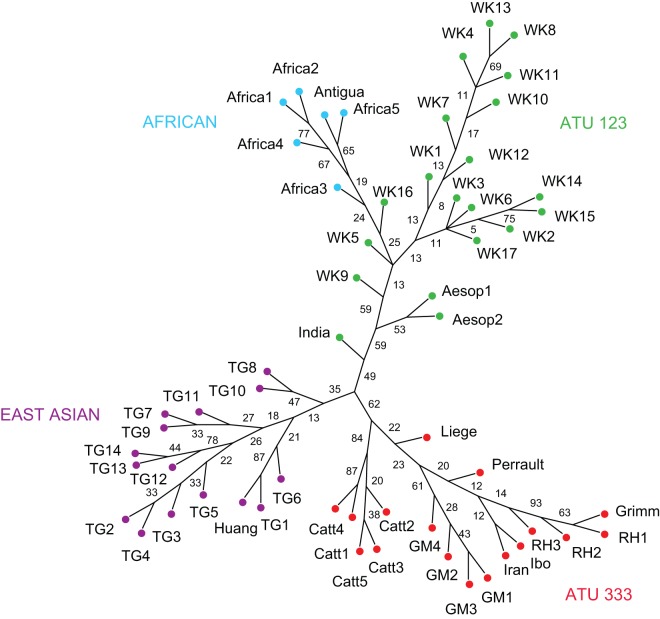
Majority-rules consensus of the most parsimonious trees returned by the cladistic analysis of the tales. Major groupings are labelled by region or ATU international type and indicated by the coloured nodes. Sub-types are indicated in the taxa labels (RH  =  Little Red Riding Hood; GM  =  Story of Grandmother; Catt  =  Catterinella; WK  =  The Wolf and the Kids; TG  =  Tiger Grandmother). Variants by particular authors, or from countries/ethnic groups that are discussed in the text have individual labels. Numbers beside the edges represent the level of support for individual clades returned by the bootstrap analysis.

The cladistic analysis returned 5740 equally most parsimonious trees (MPTs). The fit between the data and the trees was measured with the Retention Index, which was calculated as 0.72. Figure 2 shows a consensus tree representing relationships that were present in the majority of the MPTs and levels of support for them returned in a bootstrap analysis. The tree, which is unrooted, splits the tales into three principal groups. The first group corresponds to international type ATU 333, which was present in 62% of the cladograms generated from the bootstrap replicates. The group comprises the 11th century Liège tale and three recognised sub-types of ATU 333: variants of Catterinella (with bootstrap support of 84%), variants of The Story of Grandmother (61% bootstrap support), and variants of the familiar tale Little Red Riding Hood (20% bootstrap support). The latter include two non-European tales, one from Iran, the other collected from the Ibo of Nigeria. The analyses separated Catterinella from the other tales, and suggest that the 11th century Liège tale diverged from the lineage leading to Little Red Riding Hood before the latter split from the oral tale The Story of Grandmother. The Little Red Riding Hood clade separates Perrault's classic version from more recent versions, including the Grimms' 18^th^ century German text. However, the low levels of boostrap support indicate a substantial degree of conflicting signal surrounding these relationships. The second major group can be identified as international type ATU 123. This group is less well supported than the ATU 333 group, being present in only 49% of the cladograms generated from the bootstrap analysis (although it was present in all of the MPTs returned by the original analysis). The first split (with bootstrap support of 59%) in this lineage separates the Indian tale of the Sparrow and the Crow from the others. The remaining tales split into two lineages, one leading to a pair of Aesopic fables (53% bootstrap support), the other leading to the folktale The Wolf and the Kids (59% boostrap support). The latter includes a clade comprising the African tales, together with a tale recorded in Antigua (24% boostrap support). The third major group is formed by the East Asian tales. This group was the least well supported in the bootstrap analysis (35%), and does not appear to contain any robust sub-groups larger than two taxa.

**Figure 3:**
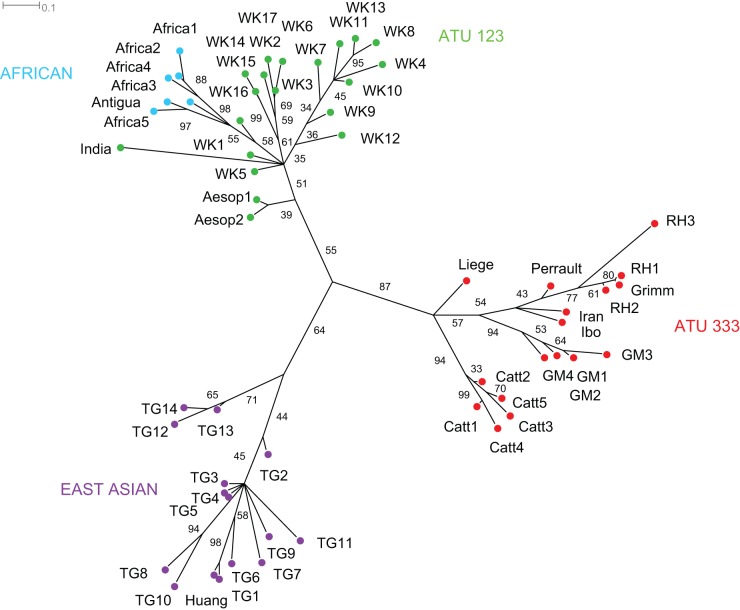
Maximum clade credibility tree returned by the Bayesian phylogenetic analysis of the tales. Major groupings are labelled by region and/or ATU international type and indicated by the coloured nodes. Numbers beside the edges represent the percentage of trees in the Bayesian posterior distribution of trees in which a given node occurred. The scale bar indicates the average number of changes per character along a given edge.

The Bayesian analysis returned a very similar set of results. Figure 3 shows an unrooted maximum clade credibility tree obtained from the posterior distribution. It represents the same three major groupings, with varying levels of support in the posterior distribution of trees. The ATU 333 group is again the most strongly supported, being present in 87% of the tree sample. Tales within this group cluster into the same recognised sub-types of ATU 333 that were returned in the cladistic analysis, including Catterinella (with posterior support of 94%), The Story of Grandmother (94%) and Little Red Riding Hood (54%), with the Liège tale forming a separate branch. Compared to the ATU 333 group, support for the ATU 123 group is relatively modest at 55%. Relationships within the group separate variants of the Aesopic fable from the other narratives. The latter clade (51% posterior probability) includes European and Middle Eastern variants of The Wolf and the Kids, the Indian tale of the Sparrow and the Crow, and a clade comprising the African tales (55% posterior probability). The final major grouping consists of the East Asian tales, which has a posterior probability of 64%. Relationships within this group generally lack resolution, except for one clade that clusters two tales from Korea (TG12 and TG13) with one from Myanmar (TG14) (71% posterior probability).

**Figure 4:**
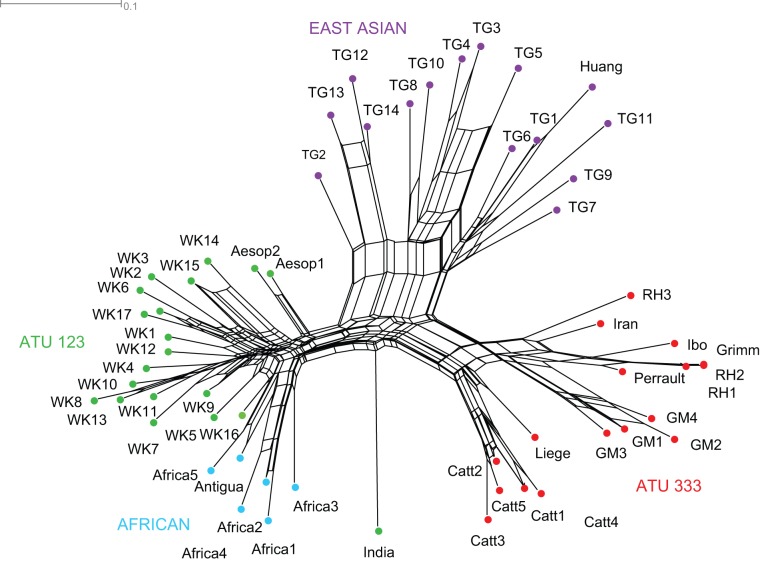
Split graph returned by the NeighbourNet analysis of the tales. Major groupings are labelled by region and/or ATU international type and indicated by the coloured nodes. Overlapping box-like structures indicate conflicting signal in the data. The scale bar indicates the proportion of characters in which states differ among the tales being compared.

The NeighbourNet graph is shown in Figure 4. Once again, the tales are divided into the same three main groups, except the Indian tale, which does not cluster with any of them. Although the groups are clearly discernible, the overlapping boxes demonstrate conflicting splits in the data. This is especially clear in the East Asian clade, which exhibits a highly reticulated structure. Similarly, overlapping boxes obscure the phylogenetic structure within the ATU 123 group, although it is possible to identify a split between the fable and folktale versions of the story, with the latter again including a clade of African tales (plus the Antiguan variant). The ATU 333 group, meanwhile, divides into two relatively well defined branches, one comprising variants of Catterinella and the medieval Liège tale, the other variants of Little Red Riding Hood and The Story of Grandmother (which each forming a distinct clade). Estimates of the overall tree-likeness/boxiness of the network yielded an average delta score of 0.3 and Q-residual score of 0.03.

## Discussion

Comparative cross-cultural studies of folklore have long been dogged by debates concerning the durability and integrity of oral traditions. While proponents of the historic-geographic approach have suggested that similar tales from different cultures can be grouped into distinct “international types” based on common origins, critics have insisted that folktales are too fluid and unstable to be classified into groups based on descent [14]. To address this problem, the present study employed three methods of phylogenetic reconstruction together with several techniques for quantifying the relative contributions of descent versus other processes in generating relationships between Little Red Riding Hood and other similar tales from around the world.

Overall, the results demonstrate a high degree of consistency in the groupings returned by the cladistic, Bayesian and NeighbourNet analyses. The “treelikeness” of these traditions appears to be relatively strong compared to other datasets. The RI of the most parsimonious trees (0.72) returned by the cladistic analysis is higher than the mean RI of both the cultural (n = 21, mean  = 0.59) and biological (n = 21, mean  = 0.61) datasets analysed by Collard et al. [47]. Simulations of cultural evolutionary processes carried out by Nunn et al. [48] suggest that datasets that return RIs of 0.60 and above are likely to have been mainly generated by branching phylogenesis. The average delta score (0.3) and Q-residual (0.03) of conflicting signal among taxa measured on the NeighbourNet graph also suggest that the data are quite tree-like. These figures are within the range of values obtained from linguistic cognate vocabulary sets reported in Gray et al. [36], and are actually lower (i.e. more tree-like) than typological features. However, it is worth noting that it is possible to obtain lower values than the ones reported here from datasets that include known borrowings and even hybrid languages. For example, Gray et al. report that a splits graph of 12 Indo-European languages based on data including loan words and the creole language Srnan yielded an average delta score of 0.23 and a Q-residual of 0.03. In other words, while relationships among the folktales fit a branching model of descent quite well, borrowing and blending could have potentially played a more significant role than indicated by the RI of the MPTs. This would be consistent with the low bootstrap support and posterior probabilities for some of the clades returned by the cladistic and Bayesian analyses. Like the NeighbourNet graph, both these analyses indicate conflicting signal surrounding the East Asian group, as well as among geographically proximate variants of ATU 333 and ATU 123.

However, it is important to emphasise that even when there is a substantial degree of blending and/or convergence among lineages, it is still possible to reconstruct robust cultural phylogenies [48], [49]. In this case, the accuracy of the relationships depicted in Figures 2, 3, and 4 is supported by qualitative evidence regarding the historiography of the tales. Thus, all three analyses identified Little Red Riding Hood, The Story of Grandmother and Catterinella as a single tale type that is distinct from The Wolf and the Kids, which folklorists believe to be a more distantly related tale [11]. In accordance with the chronological record, relationships within the ATU 333 group indicate that Little Red Riding Hood and the Story of Grandmother are descended from a common ancestor that existed more recently than the last ancestor they share with the 11^th^ century Liège poem, [40]. The position of the Grimms' version of Little Red Riding Hood supports historiographical evidence that it is directly descended from Perrault's earlier tale (via a literate informant of French Huguenot extraction) [37]. The results of the analyses also concur with the literary record on The Wolf and the Kids, which suggests the tale evolved from an Aesopic fable which was first recorded around 400 AD [46]. All three analyses indicate that Aesopic versions of the tale – in which the victim sees through the villain's disguise before letting him through the door – diverged at an early point in the history of the lineage, prior to the existence of the last common ancestor shared by other variants of The Wolf and the Kids. In sum, the consistency of the relationships returned by different phylogenetic methods, their fit to the data, and their compatibilities with independent lines of folklore research provide compelling evidence that – contrary to the claim that the vagaries of oral transmission are bound to wipe out all traces of descent in folktales – it is possible to establish coherent narrative traditions over large geographical distances and historical periods.

While these findings broadly support the goals of historic-geographic approaches to folklore, they also demonstrate that phylogenetic analysis can help resolve some the problems arising from more traditional methods. As mentioned previously, one of the key problems with existing folklore taxonomy is that it defines international types in reference to European type specimens on the basis of just a few traits. In this case, African and East Asian tales are grouped with Little Red Riding Hood because they feature human protagonists, and with The Wolf and the Kids because the villain attacks the victims in their own home, rather than their grandmother's. The phylogenetic approach used here, on the other hand, defines types in reference to the tales' inferred common ancestors rather than any existing variants, and uses all the traits they exhibit as potential evidence for their relationships. This approach yielded clear evidence that the African tales are more closely related to The Wolf and the Kids than they are to Little Red Riding Hood. All the analyses clustered the African stories with ATU 123. The sole exception was an Ibo tale, which grouped with European variants of Little Red Riding Hood, thus endorsing the collector's belief that the story is not of local origin, but an Ibo oral translation of the western fairy tale [50]. The other African tales, on the other hand, seem to have been derived from the European/Middle Eastern tale of The Wolf and the Kids, perhaps as a result of trade or colonialism. The tale was subsequently modified to create a novel redaction that spread across central and southern societies on the continent, and even as far as Antigua. Although bootstrap and posterior support for this clade was relatively modest, it is remarkable that the phylogenetic signal in this tradition was sufficiently strong to be detected by all three analyses, despite the massive cultural and human upheavals that occurred during the forced displacement of African populations during the slave trade.

The East Asian tales, meanwhile, did not cluster with ATU 333 or ATU 123, but formed a separate group. Since there is no evidence to suggest they share a more recent common ancestor with The Wolf and the Kids or Little Red Riding Hood, they cannot be classified as members of either international type. One intriguing possibility raised in the literature on this topic that would be consistent with these results is that the East Asian tales represent a sister lineage that diverged from ATU 333 and ATU 123 before they evolved into two distinct groups. Thus, Dundes has proposed that the East Asian tradition represents a crucial “missing link” between ATU 333 and ATU 123 that has retained features from their original archetype [38]. A more detailed exposition of this theory has been set out by the Sinologist Barend J. ter Haar [51]. Noting that the The Tiger Grandmother encompasses a spectrum of more ATU 333-like variants and more ATU 123-like variants, Haar argues that the East Asian tales represent an ancient autochthonous tale type that is ancestral to the other two. On the basis of qualitative comparisons among these and other Asian tales, he conjectures that the tale originated in China and spread westwards to the Middle East and Europe between the twelfth and fourteenth century, a period during which there were extensive trade and cultural exchanges between east and west. At some unspecified later point, the tale type split into the lineages that gave rise to Little Red Riding Hood and the Wolf and the Kids.

Although it is tempting to interpret the results of the analyses in this light, there are several problems with this theory. First, the earliest known version of the East Asian tale was recorded sometime in the early eighteenth century by the Chinese writer Huang Zhijun [52], thirteen hundred years after the publication of the earliest Aesopic version of ATU 123 [46], and eight centuries after the medieval variant of ATU 333 was written in Liège [40]. Of course, as mentioned previously, literary evidence about the origins of oral tales can be unreliable and biased toward Europe. However, at the very least, the existence of ATU 123 in first century Europe means that the putative Asian ancestral tale type would have to had to have spread west long before the opening of trade routes in the twelfth to fourteenth centuries, as suggested by Haar [51]. Second, if ATU 333 and ATU 123 are more closely related to each other than they are to the East Asian tales, they would be expected to share derived characters (i.e. novel story traits) that would have evolved after they diverged from the East Asian tradition. However, there is not a single characteristic shared by these two tale types that does not also occur in the East Asian group. Third, there is little evidence to support the contention that resemblances between the East Asian tales and ATU 333 and ATU 123 are primitive. If that were the case, we would expect earlier versions of ATU 123 and ATU 333 to be more similar to the East Asian tales than later variants, as original elements of the story would be lost or substituted as each tradition evolved. However, this prediction is contradicted by the available chronological data on the tales' histories. For example, some Chinese tales feature an episode that occurs in many versions of The Wolf and the Kids in which the children, suspecting that the villain may not really be their mother/grandmother, ask him to show them his hand through the door before letting him in. In ATU 123, this test first appears in a version of the fable recorded in the fourteenth century [46], and is lacking in the original version. Similarly, in Japanese, Korean and some Chinese tales the villain drinks oil/spring water to clear his throat after his initial attempts to impersonate the children's mother's voice failed. An almost identical episode occurs in variants of The Wolf and the Kids (and is also present in the African tales), in which the wolf drinks something or cuts his tongue to smooth out his voice. However, it does not appear in any recorded versions prior to the publication of the Grimms' Children and Household Tales in 1812 [1]. Similarities between the East Asian tales and ATU 333 are similarly lacking in the earliest variant, the medieval poem from Liège. They include the famous dialogue in which the victim (s) questions the “grandmother” about her strange appearance (“What big eyes you have!”), the rescue by a passing woodcutter, and the victim's escape, in which she tricks the villain into letting her go outside to go to the toilet. The latter trait has excited particular interest among folklorists, since it occurs in the oral tale The Story of Grandmother and not in Little Red Riding Hood (where the girl gets eaten). The presence of this same episode in the East Asian tradition has been cited as one of the main pieces of evidence that The Story of Grandmother is a more archaic version of the tale than Perrault's, making its absence in the Liège tale all the more conspicuous.

Bearing in mind the limitations of relying on chronological evidence about the evolution of folktales, we should consider the possibility that neither the Liège tale, nor the Aesopic fable, provide accurate representations of the early forms of ATU 333 and ATU 123, leave alone their last common ancestor. To investigate the evolution of these similarities more rigorously, the ancestral states of the traits discussed above were reconstructed on the tale phylogenies (see Methods for details). The results are shown in Table 1 below. The analyses indicate that the aforementioned similarities between the East Asian tales and ATU 333 and ATU 123 were highly unlikely to have been present in the putative archetype shared by the latter two groups, contradicting the hypothesis that the East Asian tales provide a “missing link” between the two traditions.

**Table 1:** Reconstructed ancestral states for traits shared between East Asian tales and ATU 123 and ATU 333.

	Distribution (n tales)			Ancestral in LCA of ATU 333/123	
*Trait* (character number)	East Asian	ATU 123	ATU 333	Parsimony (% of MPTs)	Bayesian (average probability in posterior tree sample)
Voice operation (27)	2	10	0	0	0.2
Hand test (30)	8	10	0	0	0.2
Dialogue with the villain (32)	7	0	10	0	0.34
Rescue by passer-by (45)	2	0	7	0	0.0
Excuse to escape (47)	9	0	3	0	0.0

To test whether key similarities between the East Asian tales and ATU 123 and ATU 333 are ancestral, the presence/absence of each trait in the hypothesised last common ancestor (LCA) of ATU 123 and ATU 333 was reconstructed under parsimony and Bayesian inference. The cells in the Parsimony column show that the traits were absent in the LCA of ATU 333 and ATU 123 in all of the most parsimonious trees (MPTs) returned by a cladistic analysis. The cells in the Bayesian column show the mean probability of each trait being present in the LCA of ATU 333 and ATU 123 across the Bayesian distribution of trees. The variance in the mean values for all traits was less than 0.1. See Methods section for details of both analyses.

An alternative – and, to the best of this author's knowledge, novel – explanation for the relationship of the East Asian tales to ATU 333 and ATU 123 is that the former is derived from the latter two, rather than vice versa. This would mean that The Tiger Grandmother represents a “hybrid” tale type, which evolved by blending together elements from ATU 333 and ATU 123 type tales. This hypothesis would account for the finding that important traits shared by the East Asian tales and Little Red Riding Hood and The Wolf and the Kids are not ancestral, suggesting that they were borrowed instead. Given the number and striking nature of these resemblances, it seems unlikely that they could have evolved independently. Borrowing is also consistent with patterns of conflicting signal in the NeighbourNet graph, which appear to be especially prevalent around the East Asian group. This impression is confirmed by a comparison of taxon-specific delta scores and Q-residuals, which are higher on average for the East Asian tales than other tales. The average delta score of the East Asian tales is 0.31 compared to an average of 0.28 for the other taxa, while their average Q-residual is 0.04 compared to 0.02. To investigate this hypothesis further, another set of analyses were carried out in which the East Asian tales were removed from the data (along with the characters that were only present in this group). It was reasoned that if these tales evolved by blending together elements of ATU 333 and ATU 123 then their removal should result in a more phylogenetically robust distinction between these two groups. This prediction was tested by maximum parsimony bootstrapping and Bayesian inference. For reference, consensus trees derived from both analyses are presented in the Supporting Information, together with a NeighbourNet graph excluding the East Asian tales (Figures S1, S2 and S3). Bootstrap support for the clade separating ATU 333 from ATU 123 increased from 62% to 83%, while the Bayesian posterior probability rose from 87% to 98%. Thus, both analyses indicate that the East Asian tales are a source of conflicting signal in the data, in line with the hybridisation hypothesis.

While on current evidence this appears to be the best available explanation for the relationships between the East Asian group and ATU 333 and ATU 123, questions remain about how, where and when the latter two tale types were adopted and combined. Based on the similarities described above, it seems likely to have occurred sometime between the origin of the lineage leading to Little Red Riding Hood and The Story of Grandmother, but before the publication of Perrault's classic tale in 1697. Shortly after this, Huang Zhijun published the first known version of The Tiger Grandmother [52], which shares elements in common with The Story of Grandmother (such as the “toilet excuse” to escape the villain), but lacks any of the features specifically associated with Little Red Riding Hood (e.g. the girl with the red hood, her being devoured by the villain, etc.), suggesting he is unlikely to have been influenced by Perrault. Given the antiquity and wide geographic diffusion of The Wolf and the Kids, it is certainly plausible that ATU 123 would have also reached China by this time, perhaps between the twelfth and fourteenth centuries, i.e. period of east-west cultural exchanges discussed by Haar [51]. Given the current state of the evidence, such scenarios are necessarily speculative. However, the digitisation and translation of an ever increasing number of folklore collections from Asia, as well as other regions, promise to yield a wealth of new data with which to investigate these questions more thoroughly in the future.

In the meantime, this case study has shown that phylogenetic methods provide powerful tools for analysing cross-cultural relationships among folktales that can be used to classify groups based on common ancestry, reconstruct their evolutionary histories, and identify patterns of contamination and hybridisation across traditions. While these goals are clearly of crucial importance to comparative studies of folklore, they also have potentially exciting applications in other fields too. As previous researchers have pointed out, the faithful transmission of narratives over many generations and across cultural and linguistic barriers is a rich source of evidence about the kinds of information that we find memorable and motivated to pass on to others [9] [53] [54]. In the present case, stories like Little Red Riding Hood, The Tiger Grandmother and The Wolf and the Kids would seem to embody several features identified in experimental studies as important cognitive attractors in cultural evolution. These include “minimally counterintuitive concepts” (e.g. talking animals) [54], “survival relevant information” (e.g. the danger presented by predators, both literal and metaphorical; the importance of following a parent's instructions, etc.) [9] [55], and “social information bias” (e.g. trust, kinship relationships, deception and false belief, etc.) [56]. Phylogenetic inference and ancestral state reconstruction methods, such as those used here, provide valuable techniques for investigating the magnitude of these biases in preserving and/or distorting narratives over long periods of time using real-world data. Equally, these methods could be applied to explore how tales are influenced by cultural, rather than psychological, selection pressures. Such an analysis might address whether local modifications of different tale-types exhibit consistent patterns, and see if they covary with specific ecological, political or religious variables. Future work on these questions promises to generate important insights into the evolution of oral traditions, and open new lines of communication between anthropologists, psychologists, biologists and literary scholars.

## Methods

### Phylogenetic Reconstruction

Cladistic analysis employs a branching model of evolution that clusters taxa on the basis of shared derived (evolutionarily novel) traits. Using the principle of parsimony, it involves finding the tree that minimises the total number of character state changes required to explain the distribution of character states among the taxa, known as the “shortest length tree” or “most parsimonious tree”. To search for the most parsimonious tree (MPT), the present analysis employed an efficient tree-bisection-reconnection algorithm implemented by the heuristic search option in PAUP 4 [57], carrying out 1,000 replications to ensure a thorough exploration of tree-space. The fit between the data and the MPTs was assessed using the Retention Index (RI) and maximum parsimony bootstrapping. The RI is a measure of how well similarities among a group of taxa can be explained by the retention of shared derived traits on a given tree [34]. A maximum RI of 1 indicates that all similarities can be interpreted as shared derived traits, without requiring additional explanations, such as losses, independent evolution or borrowing. As the contribution of these latter processes increase, generating similarities that conflict with the tree, the RI will approach 0. Maximum parsimony bootstrapping is a technique for measuring support for individual clades [58]. It involves carrying out cladistic analyses of pseudoreplicate datasets generated by randomly resampling characters with replacement from the original matrix. Support for the clades returned by the original analysis is then estimated by calculating the frequency with which they occur in the most parsimonious trees obtained from the pseudoreplicates. The bootstrap analyses reported here were carried out in PAUP 4 [57] using heuristic searches of 1,000 replicates.

Bayesian inference proceeds by calculating the likelihood of the data given an initially random tree topology, set of branch lengths and model of character evolution, and iteratively modifies each of these parameters in a Markov Chain Monte Carlo (MCMC) simulation. Moves that improve the likelihood of the data are always accepted, while those that do not are usually rejected (although some may occasionally be accepted within a certain threshold so as to avoid getting trapped in local optima). Following an initial “burn in” period, the likelihood scores will plane out and parameters will fluctuate between similar values, at which point trees are sampled at regular intervals to generate the “posterior distribution”. Unlike the trees output by a cladistic analysis, which are based on a single optimality criterion (i.e. parsimony), the posterior distribution of trees represents a set of phylogenetic hypotheses that explain the distribution of character states among the taxa under a range of plausible evolutionary assumptions. The posterior distribution of trees can be summarised by a consensus tree or “maximum clade credibility tree”, while posterior probabilities for individual clades are calculated based on their frequency in the tree sample. The Bayesian approach has been found to be particularly effective when there is wide variance in the amount of evolution that has occurred in different regions of the character data or tree, since it explicitly incorporates these parameters (i.e. branch lengths and substitution model) into the analysis [59]. The Bayesian analyses reported here were carried out in MrBayes 3.2 [60] using the model settings for “standard” (morphological) data, with the character coding set to “variable” and variance in rates of character evolution estimated under a gamma distribution. Two analyses were carried out simultaneously, each using four MCMC chains that were run for 1 million generations. Trees were sampled every 1000 generations to avoid autocorrelation, with the first 25% of the sample discarded as burnin. Log likelihood values for the remaining trees in each sample were then graphed as a scatterplot to check that the two runs had converged.

As with the other two methods, NeighbourNet clusters taxa into hierarchically nested sets. However, unlike cladistics and Bayesian inference, it does not employ a strict branching model of descent with modification, and as such these sets can overlap and intersect with one another. Accordingly, it is claimed that NeighbourNet is better able to capture conflicting signal in a dataset resulting from borrowing and blending among evolutionary lineages [35]. The method involves calculating pairwise distances between the taxa based on the character data, and generating a series of weighted splits that are successively combined using an agglomerative clustering algorithm. Relationships among the taxa are represented by a network diagram, or “splits graph”, which shows groupings in the data and distances separating them. Where the splits are highly consistent, the diagram will resemble a branching tree-like structure. Incompatible splits, on the other hand, produce box-like structures that lend a more latticed appearance to the network. The extent of reticulation in the folktale network was quantified using the delta-score and Q-residual score [36], [61]. Both measures calculate conflicting signal by comparing path lengths among pairs of taxa on “quartets” (subsets of four taxa) selected from the network. Quartets are scored from 0 to 1 according to how resolved the splits between each pair of taxa are, with values closer to 0 being more tree-like and values closer to 1 more reticulate. The estimation of the delta score includes a normalisation constant, whereas Q-residuals had to be normalised by rescaling all between-taxa distances in the network so that they average 1. The NeighbourNet analysis and calculation of d-scores and Q-residulas were carried out in SplitsTree v4.13 [35].

### Ancestral state reconstruction

Character states were reconstructed in the putative last common ancestor of ATU 123 and ATU 333 tales through parsimony analysis and Bayesian inference. In the parsimony analysis, the most parsimonious trees (MPTs) from the cladistic analysis were re-rooted so as to make ATU 123 and ATU 333 monophyletic, with the East Asian group forming a sister clade. Next, the evolutionary history of each character was reconstructed on the MPTs by minimising the total number of changes required by each tree. The ancestral state inferred for the last common ancestor of ATU 123/333 tales was then recorded for each tree. The parsimony analyses were carried out in the software program Mesquite, using the Character Trace module [62]. In the Bayesian analysis, phylogenetic relationships among the taxa were reconstructed using a topological prior that forced ATU 333 and ATU 123 to be monophyletic (making the clade present in 100% of the posterior distribution of trees). The analysis was carried out in MrBayes 3.2 [60], with the other model settings being the same as those used in the original analysis, in which the evolutionary rate across characters was allowed to vary under a gamma distribution. Estimated ancestral states in the last common ancestor of ATU 123/333 were sampled every 1000 generations to avoid autocorrelation, with the first 25% of the sample discarded as burnin. The average probabilities for each state were summarised using the Report Ancestral State command (report ancstates  =  yes), integrating uncertainty in the topological structure of the rest of the tree as well as other model parameters.

## Supporting Information

Figure S1 **Majority rules consensus of the most parsimonious trees returned by cladistic analyses of the data with East Asian tales removed.** The numbers beside the nodes represent bootstrap support values for each clade. The MPTs returned from the data had Retention Indices of 0.76.
(EPS)

Figure S2 **Maximum clade credibility tree returned by a Bayesian analysis of the data with the East Asian tales removed.** Numbers represent posterior probabilities associated with each clade.
(EPS)

Figure S3 **NeighbourNet graph of the data with East Asian tales removed.** The average delta score on the Network was 0.28 and the average Q-residual score was 0.024.
(EPS)

Table S1 **List of tales used in the analyses and their sources.**
(DOCX)

File S1 **List of characters.**
(DOCX)

File S2 **Data matrix.**
(DOCX)
