# Supplementary material for: The Phylogeny of Little Red Riding Hood
Source: PLoS One. 2013 Nov 13;8(11):e78871. doi: 10.1371/journal.pone.0078871 (PMC3827309; doi:10.1371/journal.pone.0078871)
Supplement: Table S1 — List of tales used in the analyses and their sources. (DOCX) [file pone.0078871.s004.docx]

| Taxon name | Source | Population | Map Reference | ATU type |
| --- | --- | --- | --- | --- |
| Perrault | Perrault, C. (1697). "Le Petit Chaperon Rouge" *Histoire ou contes du temps passe.* Paris: Barbin | French | 1 | 333 |
| Liege | Ziolkowski, J. (1992) A fairy tale from before fairy tales: Egbert of Liege's "De puella a lupellis seruata" and the medieval background of "Little Red Riding Hood". *Speculum:* 549-75 | Walloon | 2 | 333 |
| Grimm | Grimm J. & Grimm W. (1812). "Rotkäppchen". *Kinder- und Hausmärchen*. Berlin: Realschulbuchhandlung, no. 26 | German | 3 | 333 |
| RH1 | A. H. Wratislaw (1889) “Little Red Hood”. *Sixty Folk-Tales from Exclusively Slavonic Sources* London: Elliot Stock, pp. 97-100 | Lusatian | 4 | 333 |
| RH2 | Vasconcellos, L. (n.d.) “O Chapelinho Encarnado”. Translated by Sara Silva. Courtesy of the Instituto de Estudos de Literatura Tradicional, Lisbon. | Portugal | 5 | 333 |
| RH3 | Calvino, I. (1956, trans. 1980 by G. Martin) "The Wolf and the Three Girls". *Italian Folktales.* Harmondsworth: Penguin, pp.26-27 | Italian | 6 | 333 |
| Iran | Boulvin, A. and Chocourzadeh, E. (1975) "Un Joli Petit Paysan". *Contes Populaires Persans du Khorassan.* Paris : C. Klincksieck, pp. 110-111 | Iranian | 7 | 333 |
| Ibo | Thomas, N. T. (1913). "Little Red Riding Hood". *Anthropological Report on the Ibo-Speaking Peoples of Nigeria, Part 3.* London: Harriss & Sons, pp. 82-84. | Ibo | 8 | 333 |
| GM1 | Delarue, P. (1956). "The Story of Grandmother". *The Borzoi Book of French Folktales*. New York: Alfred Knopf, pp. 230-233. | French | 9 | 333 |
| GM2 | Joisten, C. Untitled. Recounted in Zipes, J. (1993) *The Trials and Tribulations of Little Red Riding Hood*. New York: Routledge, pp. 5-6. | French | 10 | 333 |
| GM3 | Calvino, I. (1956, trans. 1980 by G. Martin) "The False Grandmother". *Italian Folktales.* Harmondsworth: Penguin, pp.116-117 | Italian | 11 | 333 |
| GM4 | Schneller, C. (1867, trans. 2007 by D. Ashliman). "Cappelin Rosso". *Märchen und Sagen aus Wälschtirol: Ein Beitrag zur deutschen Sagenkunde.* Innsbruck: Verlag der Wagner'schen Universitäts-Buchhandlung, pp. 9-10 | Tyrolian | 12 | 333 |
| Catt1 | Schneller, C. (1867, trans. 2007 by D. Ashliman). "Cattarinetta". *Märchen und Sagen aus Wälschtirol: Ein Beitrag zur deutschen Sagenkunde.*Innsbruck: Verlag der Wagner'schen Universitäts-Buchhandlung, pp. 8-9. | Tyrolian | 13 | 333 |
| Catt2 | Calvino, I. (1956, trans. 1980 by G. Martin) "Uncle Wolf". *Italian Folktales.* Harmondsworth: Penguin, pp.49-50. | Italian | 14 | 333 |
| Catt3 | Rumpf, M. (1958) “Caterinella**:** Ein italienisches Warnmärchen,” Lago di Garda variant**.** *Fabula* 1: 76-84 | Italian | 15 | 333 |
| Catt4 | Rumpf, M. (1958) “Caterinella**:** Ein italienisches Warnmärchen**,**” Firenze variant. *Fabula* 1: 76-84 | Italian | 16 | 333 |
| Catt5 | Rumpf, M. (1958) “Caterinella**:** Ein italienisches Warnmärchen,” Serravalle variant. *Fabula* 1: 76-84 | Italian | 17 | 333 |
| Aesop1 | Perry, B. E. (400) "The She-Goat, the Kid and the Wolf". *Aesopica* vol. 1, Urbana: University of Illinois Press, no. 572, pp.614 | Unknown | Not shown | 123 |
| Aesop2 | La Fontain, J. (1668) “The Wolf, the Goat and the Kid”. *The Fables of La Fontaine (V.15)*. Translated by E. Wright. University of Adelaide (web resource) http://ebooks.adelaide.edu.au/l/la_fontaine/jean_de/fables/complete.html. Accessed 2012 June 12. | France | 18 | 123 |
| WK1 | Grimm J. & Grimm W. (1812). "Der Wolf und die sieben jungen Geißlein" *Kinder und Hausmärchen*. Gottingen, no. 5. | German | 19 | 123 |
| WK2 | Delarue, P. (1956). "The Goat and Her Kids". *The Borzoi Book of French Folktales*. New York: Alfred Knopf, pp. 300-303. | French | 20 | 123 |
| WK3 | Harvey, J. V. (1999) "The Wolf and the Kids". *The complete Russian folktale, Vol 2.* New York: Sharpe, pp. 97-99. | Russian | 21 | 123 |
| WK4 | Bruford, A. (1994) “The Grey Goat”. *Scottish Traditional Tales*. Edinburgh: Polygon, pp. 38-40. | Scottish | 22 | 123 |
| WK5 | Caballero, F. (1883). “The Carlanco”. *The Bird of Truth*. Translated by J. Ingram. Oxford: Oxford University Press, pp. 198-200. | Spanish | 23 | 123 |
| WK6 | Arslan, A. A. (1977). "Sangulum, Sungulum and Karaca Dingilim". Unpublished MA Dissertation (Story 2053), http://aton.ttu.edu/links.asp. Accessed 2011 December 10. | Turkish | 24 | 123 |
| WK7 | Lorimer, D.L.R & Lorimer, E. O. (1919) "The Story of the Wolf and the Goat". *Persian Tales*. London: Macmillan, pp. 3-5 | Iranian | 25 | 123 |
| WK8 | Sabar, Y. (1982) "The Ewe, the Goat, and the Lion". *The Folk Literature of the Kurdistani Jews*. New Haven: Yale University Press, pp. 163-166 | Iraqi Jewish | 26 | 123 |
| WK9 | Koudia, J., El. (2003) "The Mother Goat". *Moroccan Folktales*. New York: Syracuse University Press, pp. 134-135 | Moroccan | 27 | 123 |
| WK10 | El-Shamy (1999) “The Nanny Goat with the Iron Horns” *Tales Arab Women Tell and the Behavioural Patterns They Portray*. Indianapolis: Indiana University Press | Egyptian | 28 | 123 |
| WK11 | Muhawi, I & Kanaana, S. (1989) “The Little She-Goat”. *Palestinian Arab Folktales.* LA: The University of California Press, pp. 283-284 | Palestinian | 29 | 123 |
| WK12 | El-Shamy (1999) “The Mother of the Two Kids” *Tales Arab Women Tell and the Behavioural Patterns They Portray*. Indianapolis: Indiana University Press pp. 69-70 | Saudi Arabian | 30 | 123 |
| WK13 | Hangin, J. G. (1968). “The Story of the Old Sheep, the Old Goat and the Wolf” *Basic Course in Mongolian (Uralic and Altaic Series 73)*. Bloomington pp. 157-158 | Mongolian | 31 | 123 |
| WK14 | Malov, S. E. (1967) “The Ogress and Tutuqash” *Jazyk zheltyx ujgurov. Teksty i perevody*. Moscow. 107-108, no. 106 | Yugur | 32 | 123 |
| WK15 | Malov, S. E. (1967). “The Song of the Fox” *Jazyk zheltyx ujgurov. Teksty i perevody*. Moscow, 204-205, no. 198 | Yugur | 33 | 123 |
| WK16 | Lindahl et al. (1997). “The Girls and the Alligator”. *Swapping Stories: Folktales from Louisiana*. Louisiana Division of Arts, pp. 124-126. | African-American | Not shown | 123 |
| WK17 | Harris, J. (2002) “The Fire Test”. *The Complete Nights with Uncle Remus Boston:*  Houghton Mifflin Harcourt, pp. 278-282 | African-American | Not shown | 123 |
| India | Dundes, A. (1997). "The Sparrow and the Crow". *Two Tales of the Crow and Sparrow.* Lanham: Rowman & Little Field, pp. 40 - 44. | South Indian | 34 | 123 |
| Huang | Huang Zhijun (1668-1748). “The Tale of the Tiger-Woman”. Presented in Lontzen, G. (1993) “The Earliest Version of the Chinese "Little Red Riding Hood": The Tale of the Tiger-woman” *Merveilles & contes, 7* :513-527 | Chinese (Anhui) | 35 | ? |
| TG1 | Seki, K. (1966) "The Brother and Sister at Ogress' House". *Types of Japanese Folktales*. Nanzan: Asian Folklore Studies, pp 46-47. | Japanese | 36 | ? |
| TG2 | Seki, K. (1966) "Oh Sun-God, Let Down the Iron Chain!". *Types of Japanese Folktales*. Nanzan: Asian Folklore Studies, pp 46. | Japanese | 37 | ? |
| TG3 | Ikeda, H. (1971). "The Gluttonous Ogress and Children". *A Type and Motif Index of Japanese Folk-Literature*. Helsinki: Folklore Fellow Communications, pp. 90-91 | Japanese | 38 | ? |
| TG4 | Kunio, Y. (1958) “O Sun, the iron chain!”. *Japanese Folktales*. Translated by Mayer, F. Tokyo: Tokyo News Service, pp. 33. | Japanese | 39 | ? |
| TG5 | Eberhard, W. (1989) "Grandaunt Tiger". In Dundes, A. (ed) *Little Red Riding Hood: A Casebook*. Madison: University of Wisconisn Press, pp. 23-24 | Taiwanese | 40 | ? |
| TG6 | Eberhard, W. (1971) “Grandaunt Tiger” (alternative variant). *Studies in Taiwanese Folktales*. Michigan: Orient Culture Service, pp. 96-97 | Tawianese | 41 | ? |
| TG7 | Serruys, P. (1971) "The Werewolf". Fifteen Popular Tales from the South of Tatung (Shansi). *Folklore Studies 5: 210* -217. | Taiwanese | 42 | ? |
| TG8 | Falkayn, D. (2004). "Grandmother Wolf". *The Water-Buffalo and the Tiger: Folk Tales from China* (Second series). Honolulu: University Press of the Pacific, pp. 62-69 | Taiwanese | 43 | ? |
| TG9 | Wilhelm, R. (1914). "The Leopard". *Chinesische Volksmärchen*. Berlin: Jena pp. 19-22 | Chinese | 44 | ? |
| TG10 | Chiang, M (1979). “Goldflower and the Bear”. Beijing: Foreign Languages Press, pp. 1-49. | Chinese | 45 | ? |
| TG11 | Stuart, K. & Limusishiden. (1994). "The Mangus". *China's Monguor Minority: Ethnography and Folktales*. Philadephia: *Sino-Platonic Papers* 59. | Mongour Chinese | 46 | ? |
| TG12 | In-Sob, Z. (1952) "The Sun and the Moon". *Folk Tales from Korea*. London: Routledge, pp. 7-10 | Korean | 47 | ? |
| TG13 | So-un, K. (1955). "The Three Little Girls". *The Story Bag: A Collection of Korean Folk Tales.* London: Rutland, pp.76-82 | Korean | 48 | ? |
| TG14 | Maung Htin Aung. (1987). "The Sun, the Moon and the Evening Star". *Folk Tales of Burma*. New Dehlhi: Stirling, pp. 76-79. | Maynmar Hill Tribes | 49 | ? |
| Africa1 | Theal, G. M. (1886). "Demane and Demazana". *Kaffir Folk-lore.* London: Sonnenschein, Le Bas & Lowrey, pp. 118-120 | Xhosa (South Africa) | 50 | ? |
| Africa4 | Dorson, R. (1978) “A Zim Steals a Duiker's Children” . *Folktales Told Around the World*. Chicago: University of Chicago Press, pp. 400-402 | Xhosa | 51 | ? |
| Africa2 | Frazer, J. (1889). "A South African Little Red Riding Hood". *The Folk-Lore Journal 7:* 167-168 | Tswana | 52 | ? |
| Africa3 | Lambrecht, W. (1963). Number 3952 (6). *A Tale Type Index for Central Africa*. PhD thesis, University of California, Berkeley, pp.288-290. | Luba (Congo) | 53 | ? |
| Africa5 | El-Shamy (1999) “Zaynabu, ‘Omm-Zmayyim, and Their Brother’. *Tales Arab Women Tell and the Behavioural Patterns They Portray*. Indianapolis: Indiana University Press, pp. 71-72 | Sudanese | 54 | ? |
| Antigua | Johnson, J. H. (1921). "Lion Make His Voice Clear". Folklore from Antigua, British West Indies. *Journal of American Folklore,* Vol. 34: 69-70. | Antigua (Afro-Carribean) | Not shown | ? |
