# Supplementary material for: The Phylogeny of Little Red Riding Hood
Source: PLoS One. 2013 Nov 13;8(11):e78871. doi: 10.1371/journal.pone.0078871 (PMC3827309; doi:10.1371/journal.pone.0078871)
Supplement: File S1 — List of characters. (DOCX) [file pone.0078871.s005.docx]

**List of characters**†

| 1 | Species of the victim: [0] animal [1] human |
| --- | --- |
| 2 | Type of animal: [0] goat [1] rabbit [2] duiker [3] sparrow |
| 3 | Victim is [0] multiple [1] single |
| 4 | Sex of the victim: [0] male and female [1] female [2] male |
| 5 | The victim wears a red cap/hood: [0] absent [1] present |
| 6 | Relationship of the guardian to the victim: [0] mother [1] brother [2] grandmother [3] father |
| 7 | The species of the villain: [0] fox [1] ogre [2] wolf [3] tiger/leopard [4] lion [5] bush beast [6] hyena [7] bear [8] alligator [9] crow |
| 8 | The sex of the villain: [0] male [1] female |
| 9 | The relationship of the villain to the victim: [0] stranger [1] father [2] aunt/uncle [3] friend |
| 10 | The relative: [0] absent [1] grandmother [2] father [3] aunt/uncle [4] mother [5] son [6] godfather |
| 11 | The setting: [0] absent [1] woods [2] mountains [3] cave |
| 12 | Guardian builds a safe home: [0] absent [1] present |
| 13 | The child goes out: [0] absent [1] present |
| 14 | Guardian goes out: [0] no [1] to get food [2] visit relative [3] attend a feast [4] visit doctor |
| 15 | The reason for visiting the relative: [0] not stated [1] borrow a skillet the mother wants to borrow [2] illness [3] birthday [4] select son-in-law [5] birth of a child |
| 16 | The child/guardian takes food to the relative: [0] absent [1] present |
| 17 | The instruction: [0] absent [1] children are warned not to stray from the path [2] not to open door [3] not to roast meat [4] bring back cakes |
| 18 | The child eats the contents of the basket: [0] absent [1] replaces them with donkey dung [2] nails |
| 19 | Encounter with the villain en route: [0] absent [1] the child encounters villain [2] the guardian |
| 20 | Reconnaissance: [0] absent [1] villain tricks victim into coming to its house [2] villain finds out where victim is going |
| 21 | The victim and villain take separate routes [0] absent [1] take the path of needles and pins [2] the villain takes the shortcut |
| 22 | The villain tricks the relative by posing as the child: [0] absent [1] present |
| 23 | The villain kills the guardian: [0] absent [1] present |
| 24 | The villain kills the relative: [0] absent [1] present |
| 25 | The villain’s disguise: [0] absent [1] disguises as the guardian [2] disguises as the relative |
| 26 | The voice quality test 1 (villain disguises voice): [0] absent [1] present |
| 27 | Villain has an operation to clear his voice: [0] absent [1] present [2] rehearses the guardian’s voice |
| 28 | Villain clears voice: [0] sitting on ant nest [1] eats/drinks something that changes voice [2] has tongue cut [3] hot coals to burn throat |
| 29 | The password rhyme test: [0] absent [1] present |
| 30 | The hand test: [0] absent [1] present |
| 31 | The villain disguises his paws: [0] absent [1] dye [2] banana leaves to make them smooth [3] wool threads [4] rolls in dirt [5] shells |
| 32 | Victim(s) questions the villain about strange appearance: [0] absent [1] present |
| 33 | Villain replies: [0] makes excuses about the journey [1] "all the better to eat you with!" |
| 34 | Youngest sib is tricked: [0] absent [1] youngest is tricked [2] youngest warns the others |
| 35 | Villain threatens the victim ”I’m coming closer!, closer!”: [0] absent [1] present |
| 36 | The villain's tail rattles in the basket: [0] absent [1] present |
| 37 | Monster goes to bed with the victim: [0] absent [1] present |
| 38 | The monster gets into the house and all the children hide in different rooms: [0] absent [1] present |
| 39 | The monster offers grandmother’s flesh to the victim: [0] absent [1] present |
| 40 | Victim hears villain crunching bones of the sibling: [0] absent [1] present |
| 41 | Victim does not understand the warning: [0] absent [1] the villain warns her [2] the cat warns her [ |
| 42 | The villain devours the victim :[0] absent [1] yes[2] puts victim in a sack |
| 43 | Guardian comes home: [0] absent [1] present |
| 44 | The villain falls asleep after the feast: [0] absent [1] present |
| 45 | Victim rescued: [0] by guardian [1] passerby |
| 46 | The hero: [0] absent [1] huntsman [2] father [3] townsfolk [4] peddler |
| 47 | Victim tricks the villain: [0] absent [1] letting her/them outside to urinate [2] to look at neighbour’s wedding |
| 48 | Villain ties rope/intestine round the victim’s foot: [0] absent [1] present |
| 49 | Children climb to safety: [0] absent [1] a tree [2] the roof |
| 50 | Villain sees the victim’s reflection in the pond: [0] absent [1] present |
| 51 | The children trick the monster into getting into a basket and drop him: [0] absent [1] present |
| 52 | Youngest sib tells monster to get an axe to climb up: [0] absent [1] present |
| 53 | Children grease the tree: [0] absent [1] present |
| 54 | Children assisted to escape the tree: [0] Gods let down a rope to heaven [1] passerby gives tokens [2] hangs clothes on the tree to fool the villain |
| 55 | Guardian interrogates the other animals to track down villain: [0] absent [1] present |
| 56 | Guardian invites the villain for tea: [0] absent [1] present |
| 57 | Guardian gives remains of child to the villain to eat: [0] absent [1] present |
| 58 | Guardian accuses other animals: [0] absent [1] present |
| 59 | Guardian challenges villain: [0] absent [1] fight [2]game |
| 60 | Guardian bribes the judge: [0] absent [1] present |
| 61 | The villain fights with fake horns: [0] absent [1] present |
| 62 | Rescued from the villain’s stomach: [0] absent [1] cut out of the monster's belly [2] freed from bag |
| 63 | Others rescued from the villain’s stomach: [0] absent [1] Guardian [2] other people inside monster are freed [3] relative freed |
| 64 | The villain is tricked into consuming dangerous substance: [0] absent [1] drinking hot oil [1] spear [2] |
| 65 | Monster stung by bees and wasps, scorpions, etc,: [0] absent [1] present |
| 66 | Villain tricked into falling: [0] absent [1] into the fiery pit [2] river |
| 67 | The monster's belly filled with stones: [0] absent [1] present |
| 68 | Monster is transformed: [0] absent [1] tree for honey bees [2] moon [3] cabbage |
| 69 | Children transformed into stars: [0] absent [1] present |
| 70 | The villain is killed by other monsters: [0] absent [1] present |
| 71 | Victim flees through the woods, and uses the help of the river, mountain, etc. to obstruct the villain’s pursuit: [0] absent [1] present |
| 72 | Victim hides until the morning, awaiting the villain's return: [0] absent [1] present |

† Traits that were either present or absent (e.g. character 24 “The villain kills the relative”), or could only take two forms (e.g. character 1 “The victim is human or animal”) were coded as binary characters. Traits that could take numerous possible forms (e.g. the species of the villain) were coded as multi-state characters, rather than as a series of binary presence/absence characters to avoid redundancy.

In cases where the potential occurrence of a particular event in the story was impossible due to events that happened or did not happen earlier, the character in question was coded as a gap (represented by the “-“ symbols in the matrix). For example, character 27 “The villain has an operation to clear his voice” depends on an earlier episode (character 26 “The voice test”) where he attempts to disguise his voice but is detected by the victim. Therefore, for tales in which the latter episode were absent, character 27 was coded as a gap. Gaps are treated as phylogenetically uninformative by the analyses, thereby avoiding the possibility of tales being grouped on the basis of traits they logically could not have.
