# Supplementary material for: The Phylogeny of Little Red Riding Hood
Source: PLoS One. 2013 Nov 13;8(11):e78871. doi: 10.1371/journal.pone.0078871 (PMC3827309; doi:10.1371/journal.pone.0078871)
Supplement: File S2 — Data matrix. (DOCX) [file pone.0078871.s006.docx]

**Data Matrix**†

Character 1 10 20 30 40 50 60 70

Grimm 1-111020011010211012110120--00-11-001-0001-1110-00----000000-1300010-000

Perrault 1-1110200110102110121101210-00-11-001-0001-0000-00----000000-00000-0-000

RH2 1-111020011010211012110120--00-11-001-0001-0110-00----000000-1300010-000

GM1 1-110020010010010012200100--00-11-001-1010-0001100----000000-00000-0-000

GM3 1-11001001001011000--00100--00-11-001-1000-0001100----000000-00000-0-010

GM2 1-111020011010010012200100--00-11-001-1020-1001100----000000-00000-0-000

GM4 1-111210010010010012200100--00-11-001-1021-0000-00----000000-00000-0-000

RH1 1-111020011010211012110120--00-11-001-0001-1110-00----000000-1300010-000

Iran 1-1200200210100100121000210-00-11-001-0001-0120-00----000000-00000-0-000

Ibo 1-121051011010210012110100--00-11-001-0000-0120-00----000000-00000-0-000

Catt2 1-11002023101011410---0000--00-0--001-0001-0000-00----000000-00000-0-000

Catt3 1-11002023101011410---0000--00-0--001-0001-0110-00----000000-11000-0-000

Catt5 1-11002023101011410---0000--00-0--001-0001-1000-00----000000-10000-0-000

Catt1 1-11001123101011410---0000--00-0--101-0001-0000-00----000000-00000-0-000

Catt4 1-11001133101011410---0000--00-0--100-0003-0000-00----000000-00000-0-000

RH3 1-110-20040010210212100110--00-01-101-0001-0140-00----000000-1300000-000

Liege 1-111-21061010-00-10-00000--00-0--000-0000-0000-00----000000-00000-0-000

TG1 1-000-1101-010010011-00020--00-0--00100101-000??100003000000-00000-0-101

Huang 1-000331012010010011-00020--00-10-00100101-00011100003000000-00000-0-101

TG10 1-00007?01000211000--000210-00-0-101100000000011110010000000-00200-0-000

TG6 1-000030010012000001-00020--00-0--00100101000012000000000000-00100-0-001

TG5 1-01003101200241002--010211101210000100101-00011110010000000-00100-0-000

TG8 1-01002101200230200--000210-00-10101100000000010101000000000-00000-0-000

TG9 1-00003001200200202--01010--00-10000100101-00020101002000000-00000-0-001

TG7 1-000020000001-1002---1010--0100-100100101-00010101002000000-00000-3-000

TG11 1-00001105200251202--01020--0140-100100101-01511101000000000-00000-0-010

TG4 1-000011000003-00-2---1010--0120-001100101-00011110111000000-00100-20000

TG3 1-000011012002-00-2---1011110120-000000101-00011110111000000-01100020000

TG2 1-000011000001-02-2---1010--00-0-000?00001-000??110101000000-00000-20000

TG12 1-000231052003-1002---10110-01010000000000-0000-110111000000-00000-01000

TG13 1-01003?002001-12-2---10110-01010000000000-0000-110111000000-00000-21000

TG14 1-010031000003-12-2---10110-01010100000000-0000-100111000000-00000-01000

Aesop1 00120020000001-02-0---00112-00-0-00000000000000-00----000000-00000-0-000

Aesop2 00120020000001-02-0---0010--1100-00000000000000-00----000000-00000-0-000

WK1 000?0020000001-02-0---0011111110-00001000111200-00----000000-1000010-000

WK4 000?0000000001-02-0---0010--0130-00000000111200-00----100100-1000000-000

WK17 010?0320000001-02-0---00112-10-0-00000000110200-00----010020-00001-0-000

WK3 000?0020000001-02-0---00111210-0-00001000110200-00----010020-00001-0-000

WK9 000?0020000001-02-0---00112-00-0-00000000110200-00----000110000000-0-000

WK7 000?0020000001-02-0---0010--0110-00001000110200-00----000111100002-0-000

WK10 000?0020000001-02-0---0011121110-00001000110200-00----10011011000000-000

WK11 000?0060000001-02-0---0010--1100-00000000010200-00----00011011000000-000

WK12 000?0001000001-02-0---0010--1140-00001000110200-00----000010000000-0-000

WK2 000?0020000004-00-2-0-00111210-0-00001000111200-00----010000-1000100-000

WK6 00000020000001-02-0---0011111110-20001000110000-00----010020-00001-0-000

WK13 00000020000101-00-0---00100-1150-00000000110200-00----10011011000000-000

WK8 000?0040000101-00-0---00110-1110-00000000110200-00----10011111000000-000

WK14 1-120011000001-00-0---00110-10-0-00001000110200-00----001000-00001-0-000

WK15 00120020000001-00-0---00110-10-0-00001000110200-00----001000-00001-0-000

WK5 000?00?0000001-02-0---00112-10-0-00000000010200-200---000000-0001000-000

Africa1 1-110110003001-03-0---00111310-0-00000000210200-00----000000-20010-1-000

Africa2 1-110010000001-02-0---00111310-0-00000000210200-00----000000-20010-1-000

Africa3 1-120010103001-02-0---00112-?0-0-00000000110200-00----100000-1200000-000

Africa5 1-010160000001-00-0---00111010-0-00000000110200-00----110000-1200000-000

Antigua 1-110140000001-00-0---00111310-0-00000000110200-00----110000-00000-0-000

Africa4 02100010000001-02-0---00111310-0-00000000210200-00----120000-20000-1-000

WK16 1-010080000001-02-0---00112-10-0-10000000110200-00----000000-1000000-000

India 03000091300100-00-----0000--00-0-00010010100200-00----010000-1010000-000

†Values refer to character states described in File S1 ‘List of Characters’. Dashes represent gaps (i.e. uninformative character states – see File S1 for detailed explanation).
